# Bevacizumab in Pediatric Neuro-Oncology

**DOI:** 10.3390/curroncol32100573

**Published:** 2025-10-16

**Authors:** Jacob Silverman, Sayanthen Sathyakumar, Hallie Coltin, Sebastien Perreault, Nada Jabado, Eric Bouffet, Samuele Renzi

**Affiliations:** 1 McGill Faculty of Medicine and Health Sciences, Montréal, QC H2V 4A9, Canada; 2 Sunnybrook Research Institute, University of Toronto, Toronto, ON M4N 3M5, Canada; 3 Division of Hematology-Oncology, CHU Sainte-Justine, Université de Montréal, Montréal, QC H3C 3A7, Canada; 4 Department of Neurology, Division of Child Neurology, CHU Sainte-Justine, Université de Montréal, Montréal, QC H3C 3A7, Canada; 5 Department of Pediatrics, McGill University, Montreal, QC H4A 3J1, Canada; 6 The Research Institute of the McGill University Health Centre, Montreal, QC H4A 3J1, Canada; nada.jabado@mcgill.ca; 7 Division of Pediatric Hematology-Oncology, Hospital for Sick Children, Toronto, ON M5G 1E8, Canada; eric.bouffet@sickkids.ca; 8 Department of Pediatrics, Division of Hematology-Oncology, CHU de Québec, Université Laval, Québec City, QC G1V 4G2, Canada; samuele.renzi.med@ssss.gouv.qc.ca

**Keywords:** bevacizumab, pediatric neuro-oncology, angiogenesis inhibitors

## Abstract

Bevacizumab is often used off-label in pediatric neuro-oncology, and evidence for indications of bevacizumab use in pediatric neuro-oncology is often fragmented. Therefore, this review aims to provide an organized summary of efficacy across different types of tumors, highlight outcomes, and link findings to the underlying biology. Gaps in the literature were also identified to guide future research. We narratively synthesized various pediatric studies, and the following tumor categories were identified for discussion: low-grade glioma, high-grade glioma, diffuse intrinsic pontine glioma, schwannoma, medulloblastoma, radiation necrosis, and cerebral edema. Key outcomes considered included overall survival, event-free survival, progression-free survival, vision and/or hearing improvements, steroid use, quality of life, and toxicity. The greatest benefits were observed in cases such as recurrent medulloblastoma in combination with temozolomide and irinotecan, optic pathway glioma visual function, and diminished steroid use in radiation necrosis. Results were poorer in cases of newly diagnosed high-grade gliomas and diffuse intrinsic pontine gliomas. The medication was overall well tolerated, with adverse events like hypertension, proteinuria, and epistaxis often being manageable with surveillance. In consideration of the results, bevacizumab should be considered based on the tumor profile, and its outcome measured along functional endpoints, besides radiological evolution. Continued investigations into outcome measures, as well as combination with targeted treatments and optimizing therapy, will contribute to improving outcomes in this vulnerable population.

## Simple Summary

Options for children with central nervous system (brain and spinal cord) tumors are often limited. This review aims to outline different types of central nervous system tumors in children, as well as related conditions (treatment-related swelling or radiation injury), and different ways bevacizumab can be used in their treatment. Bevacizumab is an inhibitor of new blood vessel formation, thus aiming to block the blood supply to growing tumors. Often, this is in combination with other existing treatments. Results of previous studies are discussed, and their implications for treatment are noted, such as where benefits are and when results have been poorer. The outcomes discussed go beyond scans and examine other factors, including quality of life, vision, and hearing. Common side effects of the medication are clarified in the context of overall safety and toxicity. Finally, a discussion of where existing literature may lead in the future is provided.

## 1. Introduction

Brain and other nervous system tumors are the most common type of solid tumors and the second most prevalent form of cancer in the pediatric population after leukemia [1]. There have already been notable advancements in treatment for the vast diversity of tumors, resulting in increased efficacy and reduced toxicity, and treatment options continue to evolve as our understanding of the molecular biology behind these tumors deepens [2]. Pediatric brain tumors are highly heterogeneous, hence requiring specific molecular characterization and therapeutic treatments. The World Health Organization’s (WHO) Central Nervous System V (CNS5) is the most up-to-date classification of tumors, reflecting our current understanding of the biology behind these tumors [3].

Vascularization has long been considered a hallmark of cancer, although some tumors are able to grow in the absence of neovascularization [4]. Bevacizumab (Avastin) is a monoclonal antibody that selectively binds to Vascular Endothelial Growth Factor (VEGF) and inhibits associated pathways that drive neovascularization [5]. Inhibition of these pathways results in a reduction of vascular proliferation and blood supply to tumor tissues. Since its initial approval by the Food and Drug Administration (FDA) in 2004 for the treatment of metastatic colorectal cancer, bevacizumab has now been approved for several solid tumors, both in pediatric and adult settings.

Bevacizumab was first introduced in the pediatric setting in 2008 through a study evaluating the efficacy of bevacizumab in pediatric patients with recurrent or progressive solid tumors [6]. Among patients who demonstrated a partial response, two had astrocytoma grade III. The authors concluded that the treatment had a satisfactory acute safety profile, showed promising antitumor activity, and highlighted the necessity of further trials to investigate the efficacy and safety of the treatment in pediatric patients.

Here, we provide an overview of bevacizumab, including its clinical benefits, limitations, and future potential in the treatment of pediatric brain tumors. It should be noted, though, that despite the evidence provided below, bevacizumab is currently not FDA-approved in the management of these cancers.

## 2. Bevacizumab in Pediatric Low-Grade Glioma

Pediatric low-grade glioma (pLGG) is the most common form of CNS tumors in children, accounting for more than 30% of all CNS tumors in this population [7]. The most recent classification of CNS tumors (WHO 5th edition) has made significant changes to the classification of tumors because of a better understanding of their molecular and histopathological characteristics [3]. The new WHO Classification separates pLGG into three families: pediatric type diffuse low-grade, circumscribed astrocytic gliomas, and glioneural and neuronal tumors [3]. Low-grade gliomas are slow-growing and heterogeneous tumors.

The standard of care for pLGG involves surgical resection, which remains the gold standard when feasible; when surgical resection is not possible or it is incomplete, chemotherapy and (rarely) radiotherapy can be considered in an adjuvant setting. Of note, radiation therapy is generally not favored due to its adverse effects on the growing brain and risk of significant morbidities long-term [2]. Advances in the understanding of the molecular biology behind these tumors have resulted in alternative treatment options, such as targeted therapy [8].

Packer et al. conducted a retrospective review evaluating the combination of bevacizumab and irinotecan in pediatric patients with LGG. In this retrospective case-series of ten pediatric patients with multiple recurrent pLGG, the treatment regimen led to objective radiographic responses in seven (78% (one patient was deemed non-evaluable)) patients (one CR, four PR, two minor response) and clinical improvement in seven (78%) patients, including weight gain, improvements in visual acuity, reversal of psychomotor retardation, and improvement in developmental status (i.e., motor control, verbal milestones) [9].

The Pediatric Brain Tumor Consortium initiated a study (PBTC-022) evaluating the efficacy of a combination of bevacizumab and irinotecan (CPT-11) in pediatric patients with recurrent low-grade gliomas. Thirty-five patients (median age 8.4 years) received BVZ + CPT-11 every two weeks (median of 12 courses in addition to single-agent BVZ) until two years of therapy was achieved or negative events were observed, including progressive disease and significant toxicity. The results were overall underwhelming, as only two patients experienced sustained partial response, with an objective response rate (>8 weeks) of 6%. All but one patient experienced disease progression at a median of 5 months following treatment cessation, suggesting that this treatment may be beneficial only in the short term. The authors concluded that BVZ + CPT-11 may have a role in patients who failed standard chemotherapy regimens and can help in tumor control in young children to delay or avoid radiotherapy, which is known to have serious adverse effects [10]. Other reports have confirmed the feasibility of this approach [10,11].

Bevacizumab is generally tolerated in pediatric patients with low-grade gliomas, with the majority of studies reporting grade 1/2 toxicities, including hypertension and fatigue, that are usually reversible [9,10]; however, a systematic review from Lu et al. reported that an estimated 1 in 10 children experienced grade 3 toxicity or higher [12].

A nationwide evaluation of bevacizumab-based therapies (BBTs) in pLGG patients in the United Kingdom concluded that bevacizumab resulted in meaningful clinical benefit with excellent tolerability, although these effects were not sustained after discontinuation of therapy [13]. Notably, the maximum benefit was observed in patients treated at an earlier stage of the disease. Overall, partial response (PR) was noted in 40% of patients, stable disease (SD) in 49% of patients, and progressive disease (PD) in 11% of patients. The median time to best response was 3 months since initiation of treatment. Unfortunately, disease progression was seen in 65% of patients at a median time of 8 months after cessation of treatment, suggesting a short-term benefit in this cohort. Of note, 85% of patients in this cohort received BBT as a third-line treatment and still experienced benefit despite an expected decrease in efficacy after several treatments [13].

As an antiangiogenic agent, bevacizumab rapidly changes the permeability of the blood–brain barrier, and the MRI response to bevacizumab may have been overstated based on Response Assessment in Neuro-Oncology (RANO) HGG criteria that use T1+contrast sequences. This may explain why the real benefit of bevacizumab in terms of response rate has been questioned.

Finally, Levenbaum et al. highlight the lack of data on the effects of bevacizumab on cystic components of pLGG, which may enlarge, causing clinical complications. They present a case series of four patients with predominantly cystic LGG. All of them demonstrated a response to bevacizumab with reduced cyst size. Although based on a small cohort, their results suggest an important role for bevacizumab in alleviating cyst burden and its associated effects [14].

### Visual Benefit

Standard chemotherapy regimens have been shown to produce minimal visual improvements in patients with optic pathway glioma (OPG), whether NF1-related or sporadic. The use of BBT has demonstrated visual improvements in patients with OPGs and associated vision loss.

A 2013 case series from Avery et al. reported on four pediatric patients with OPG (two NF1-related and two sporadic) who received bevacizumab following progressive worsening noted in visual acuity (VA) and visual field (VF) assessments. Three patients had previously received chemotherapy including carboplatin/vincristine. All patients demonstrated significant improvements in VA, VF, or both; remarkably, one patient demonstrated complete recovery of VF deficits, while another patient demonstrated complete recovery of visual acuity. All three patients with visual acuity loss experienced visual acuity improvement ranging from 0.3 logMAR (moderate) to 0.8 logMAR (significant) [15].

These findings align with earlier reports of BBT in pediatric patients with recurrent low-grade glioma where improvements in vision were also observed. Hwang et al. (*n* = 14) noted that six children had cancer-related visual impairment. With initial bevacizumab-based therapy, 4/6 patients (67%) demonstrated visual improvement. The authors add that one patient demonstrated recurrent visual amelioration with each course of treatment [16]. Packer et al. reported improvement in visual acuity in 2/9 of evaluable patients (22%) receiving combination treatment with bevacizumab and irinotecan for recurrent LGGs [9].

In a recent single-institution retrospective study evaluating bevacizumab as a second-line treatment for pediatric patients (median age at treatment initiation of 8.1 years old) with unresectable pLGGs, all patients showed no signs of further disease progression at the 3-month mark. Approximately one-third of patients showed radiological and visual improvement, while two-thirds of patients had SD. Most notably, among the patients with visual impairment but radiological stability at the end of treatment, 71% had an NF1 diagnosis. While progression-free survival (PFS) was noted to be 91.4% at 12 months, it reduced to 31.4% at the 36-month mark [17].

In a recent study evaluating bevacizumab as a single-agent treatment for OPG in 31 patients under 19 years of age, ophthalmologic disease control was achieved in 88% of patients [18].

Similarly, in a nationwide multi-institutional study conducted in the Netherlands, pediatric patients with OPG who received bevacizumab demonstrated favorable short-term outcomes [19]. Most patients experienced temporary stabilization in tumor volume and improvement in visual function. Interestingly, 64% of patients experienced radiological tumor progression after a median of 20 months since initiating therapy; however, 74.4% of eyes evaluated had stable visual acuity, and 73% of visual fields improved, this being in keeping with the known poor correlation between radiological and clinical findings in pediatric patients with OPG [13]. Interestingly, among patients of this cohort, those with KIAA1549-BRAF fusion had a lower PFS compared to those with NF1-OPG.

Finally, the final results of OZM-063, a clinical trial examining combination vinblastine and bevacizumab compared to vinblastine alone in children with unresectable or progressive LGG, are awaited. In this trial, response rate at 6 months is the primary outcome measure, and PFS, 5-year OS, and visual improvement are among the secondary outcome measures [20]; preliminary results suggest that adding bevacizumab to vinblastine could result in a better radiological response, as well as potential visual benefit in pediatric patients with low-grade glioma [21]. Of note, bevacizumab has often been used as a second-line treatment or later; however, data from the OZM-063 study and the UK study by Green et al. [13,20] suggest that the use of bevacizumab at an earlier stage may prove more beneficial, particularly for patients with impaired vision [13,20].

## 3. Bevacizumab in High-Grade Glioma

Pediatric High-Grade Gliomas (pHGGs) are aggressive brain tumors that account for 8-12% of CNS tumors in children [22]. Despite advancements in treatment, the 5-year survival rate remains dismal, below 20% [23,24]. The standard of care for pHGG involves maximal safe resection followed by radiotherapy and usually adjuvant chemotherapy [25].

While bevacizumab has demonstrated promising results in adults with recurrent high-grade gliomas, its use in pHGGs has demonstrated less satisfactory results. The PBTC led a phase II study evaluating the efficacy of bevacizumab and irinotecan in pediatric patients (median age, 15) with recurrent CNS tumors, including HGG and diffuse intrinsic pontine glioma (DIPG). The combination treatment did not result in sustained objective responses [26].

The HERBY trial was a phase II, open-label, multicenter study assessing whether the addition of bevacizumab compared to standard radiotherapy plus temozolomide would improve outcomes in pediatric patients with newly diagnosed non-brainstem high-grade glioma. The trial enrolled 121 patients and found no significant improvement in event-free survival (EFS) or overall survival (OS) with the addition of bevacizumab [27]. Moreover, bevacizumab was associated with a higher rate of serious adverse events and treatment discontinuations [27]. In a post hoc analysis of the HERBY trial, Mackay et al. found that the tumors with MAPK pathway alterations demonstrated improved survival when treated with bevacizumab [28].

The Children’s Oncology Group recently reported the results of a randomized, phase II multi-center study (ACNS0822) evaluating the efficacy of concurrent vorinostat or bevacizumab with focal radiation therapy (RT) in 1-year EFS compared to standard concurrent TMZ with focal RT in pediatric patients with newly diagnosed HGG. All patients received maintenance doses of bevacizumab and temozolomide. Among ninety randomized patients, 1-year EFS rates were 43.8% for the bevacizumab arm, 41.4% for the vorinostat arm, and 59.3% for the temozolomide arm, with no statistically significant differences between arms. The authors concluded that treatment with vorinostat and bevacizumab was not superior to TMZ in this subgroup of patients [29].

Similarly, Narayana et al. conducted a study evaluating the efficacy of bevacizumab in pediatric patients with recurrent high-grade glioma, inspired by the promising outcomes seen utilizing bevacizumab in adult cohorts with high-grade gliomas. However, the results were disappointing and notably lower than those observed in adult series, with a radiographic response rate of 16.7%, a median PFS of 2.5 months, and an OS of 5.5 months [30]. The reports, however, are not unanimous regarding this subject: Detti et al. showed that bevacizumab could play a role in improving survival on the basis of their retrospective single-center analysis on 92 patients with recurrent HGG [31]. A scoping review from Fu et al. also suggested that bevacizumab seems to improve PFS as well as quality of life in patients with recurrent glioblastoma [32].

The varied results seen between adult HGG and pediatric HGG with respect to VEGF inhibition could suggest that angiogenesis in pHGG is regulated by multiple growth factors, including platelet-derived growth factor (PDGF) and fibroblast growth factor (FGF) [33].

At the Seattle Children’s, Crotty et al. retrospectively studied thirty-six children with pHGG and DIPG treated with radiotherapy followed by maintenance temozolomide, irinotecan, and bevacizumab. Molecular analysis of the tumors was also conducted when available. Survival in pHGG was comparable to results obtained using other contemporary three-drug treatment protocols. Specifically, in DIPG, 1-year OS appeared higher than when using the historical regimen on temozolomide alone. Despite these results, in pHGG, a cure was rarely observed, and in DIPG, it was non-existent. The authors therefore conclude that this could serve as a backbone for additional agents in the future [34]. Given the findings of Mackay et al. regarding improved survival in pHGG with MAPK pathway alterations [28], Crotty et al. speculate that their cohort may have contained a higher concentration of MAPK-altered tumors [34]. However, such an effect may reflect the underlying nature of pHGG with MAPK pathway alterations rather than being directly due to bevacizumab.

## 4. Bevacizumab for Diffuse Intrinsic Pontine Glioma

The median survival time of patients with DIPG has remained unchanged for decades, with focal radiotherapy as the only effective palliative treatment. The introduction of re-irradiation has contributed to extending the survival of some patients, but the overall 5-year survival of this condition remains below 5% [35]. Yet, with other types of tumors demonstrating VEGF overexpression, bevacizumab appeared to be a potential therapeutic option.

The PBTC thus began a phase II trial in children with malignant glioma and DIPG to examine if bevacizumab could be an effective treatment. Bevacizumab and irinotecan were administered to thirty-one eligible patients, seventeen of whom had DIPG. Unfortunately, no sustained responses were observed in any of the patients. The median PFS was only 2.3 months, and at 6 months, only 9.7% demonstrated PFS [26].

In 2021, El-Khouly et al. reported a phase I/II study, this time with erlotinib paired with bevacizumab and irinotecan. They reported an OS of 13.8 months, a result similar to that obtained in the Seattle Children’s Hospital’s study [34], but indeed longer than the 10 months achieved with radiotherapy alone. Interestingly, they found that their treatment regimen was especially effective in the patients stratified as intermediate or high risk (12.8 months and 18.7 months, versus 9.7 and 7.0 months) [36].

The Seattle Children’s Hospital conducted a retrospective study evaluating the efficacy of this regimen in pediatric patients with Grade III or IV high-grade glioma or DIPG. This regimen was found to be tolerable and showed a slight improvement in survival for DIPG patients compared to temozolomide alone, with an 80% survival rate at 1 year compared to 45.3% based on International DIPG registry data and 40% seen in patients treated with temozolomide alone in the ACNS0126 trial. However, survival for DIPG patients treated with this regimen decreased to 10% at the 2-year mark and to 0% at 5 years [34].

In a systematic review on the role of bevacizumab-based regimes in the treatment of DIPG, Evans et al. found conflicting conclusions among included papers in terms of median OS, radiological response, symptom improvement, quality of life, steroid use, and radiation necrosis. While the findings may suggest that the use of bevacizumab could result in an improvement in quality of life and reduced steroid use, the authors concluded that the evidence is not strong enough to generalize the results [37].

## 5. Bevacizumab for Other CNS Tumors

Bevacizumab has also shown promise in other CNS tumors, such as schwannomas, in the setting of neurofibromatosis type 2 (NF2). In a systematic review by Tops et al., the authors demonstrated that bevacizumab’s benefit on the tumor volume in children is less pronounced than in adults, with tumor regression in 11% of pediatric patients, size stability in 68%, and tumor progression in 9-22%. In contrast, in adults undergoing bevacizumab-based treatment, tumor regression was observed in 30% of patients in a meta-analysis by Shi et al. [38], and 38% of patients in a systematic review by Chiranth et al. [39]. Chiranth et al. also found tumor progression in only 9% of patients [39,40].

However, the effect of bevacizumab on hearing function is more comparable to adults’ results, with improved hearing in 33% of patients, worsened hearing in 7%, and in 60%, hearing remaining stable. Likewise, Renzi et al. demonstrated that 61% of patients with initial hearing loss demonstrated improved hearing after six months of bevacizumab. Thus, bevacizumab seems to be a promising option for NF2 in children, especially with regard to hearing preservation [41].

Gururangan et al. conducted the first efficacy study of bevacizumab in the treatment of recurrent ependymoma. Thirteen evaluable patients were administered bevacizumab and irinotecan for a median of three courses. No objective response was observed, nor was there an improvement in the rate of disease stabilization compared to standard regimens. The reason for this lack of response was not clear to the authors, and they speculate that it may be due to biologic heterogeneity of ependymomas or implication of angiogenesis factors other than VEGF [42].

The first report on the use of bevacizumab in medulloblastoma was in 2011. Aguilera et al. described two patients with recurrent medulloblastoma treated with a combination of bevacizumab, irinotecan +/- temozolomide who experienced sustained tumor progression [43]. Subsequently, in a phase II trial, Levy et al. compared the combination of temozolomide–irinotecan versus temozolomide, irinotecan, and bevacizumab in recurrent medulloblastoma or supratentorial primitive neuroectodermal tumor (spNET, an older term currently replaced by new classifications). They reported an improved median OS (13 months vs. 19 months) and median EFS (6 months vs. 9 months) with the use of bevacizumab. The results of this trial have influenced clinical practice, as the combination of irinotecan, temozolomide, and bevacizumab has now become a standard of care for patients with recurrent medulloblastoma in many institutions [44]. Unfortunately, this study was designed before the awareness of medulloblastoma subgrouping, and whether the benefit of bevacizumab is subgroup-specific was unknown. Importantly, this study did not show any advantage of bevacizumab in non-medulloblastoma (spNET) patients.

The Seattle Children’s Hospital [45] followed the protocol in the above study, concluding that their outcomes were similar to those published in clinical trials. Interestingly, they add that this treatment regimen would be especially useful in patients geographically limited from care.

An ongoing clinical trial is combining the triple therapy of temozolomide, irinotecan, and bevacizumab with cRIT 131I-omburtamab in recurrent medulloblastoma, with the latter being a medication consisting of the delivery of radiolabeled tumor-specific antibodies.

Finally, the Medulloblastoma European Multitarget Metronomic Anti-Angiogenic Trial (MEMMAT) evaluated a multi-drug regimen for the treatment of recurrent medulloblastoma. The treatment combined daily oral thalidomide, fenofibrate, celecoxib, and 21-day cycles of either oral etoposide or cyclophosphamide, in a metronomic fashion. The protocol also included intravenous bevacizumab and intraventricular therapy (etoposide and cytarabine). This unique approach aims to target the tumor microenvironment both by targeting angiogenesis directly, as well as by activating the immune system. The trial yielded promising results, with 23 (57.5%, *n*=40) of the patients achieving disease control following six months of treatment. Of the twenty-three, three (7.5%) had no evidence of disease, six (15%) had a complete response, nine (22.5%) had a partial response, and five (12.5%) demonstrated stable disease. Unfortunately, seventeen patients had to cease treatment due to disease progression [46].

## 6. Bevacizumab for Radiation Necrosis

Radiotherapy is a central part of the treatment plan for many pediatric brain tumors. Yet, despite its benefit, it may be associated with substantial side effects, including a risk of radiation necrosis [47]. The pathophysiology behind radiation necrosis is still unclear. Vellayappan et al. suggest that it involves endothelial injury and subsequent vascular changes in both healthy and tumoral tissue. Among the enumerated inflammatory markers involved in this process are VEGF, TNFα, and HIF1. As part of their hypotheses, they suggested that the fibrinoid necrosis of small vessels could lead to ischemia and necrosis of the brain tissue [48].

The implication of VEGF in this process is appealing, considering that bevacizumab is a VEGF inhibitor. Logically, if VEGF contributes to the induction of vascular permeability and consequently severe edema, such as that observed in radiation necrosis, its inhibition should provide benefit [49].

Indeed, while the mainstay of therapy for radiation necrosis remains dexamethasone, side effects and potential failure of response have left room for a more effective and tolerable treatment [47].

Several studies have investigated the use of bevacizumab in children for this purpose.

Liu et al. described four children with pontine gliomas and associated radiation necrosis. Of the four, three demonstrated significant clinical improvement with bevacizumab and were able to cease their steroids. Of note, the other patient’s failure to respond was found to be due to disease progression, rather than radiation necrosis. Thus, the authors concluded that bevacizumab may prove to be a useful agent in managing radiation necrosis [50].

Baroni et al. followed a group of twenty-six pediatric patients with suspected radiation necrosis. Upon presentation, twenty-two patients were administered a high dose of dexamethasone. Within the first two weeks following initiation of dexamethasone, every patient received bevacizumab. Eighteen patients were able to taper their dexamethasone, while thirteen demonstrated objective neurological improvement, and thirteen demonstrated radiological response. Four patients experienced progression of their disease. They additionally found that there was a good response regardless of how early or late the radiation necrosis was. Furthermore, they did not find any correlation between the dose of bevacizumab (5 or 10 mg/kg every 2 weeks) and the response. There was likewise no statistically significant predictor found for the response to bevacizumab [47].

In a systematic review, Drezner et al. [51] found that using adjuvant agents in conjunction with corticosteroids, such as bevacizumab, is safe, tolerable, and may provide a greater benefit in older children, as it was more often older patients who did not respond to steroids. More specifically, they found that (91%, *n* = 11) patients receiving bevacizumab with steroids improved both in terms of symptoms and imaging.

In a systematic review on the role of bevacizumab in treating DIPG in children, Evans et al. also addressed its role in treating radiation necrosis. Among eight children included in a study, three improved clinically, four remained stable, and one progressed [47]. Only five were assessed for radiological response, and among these five, two had a radiological decrease in the necrosis at MRI, and three were stable. Notably, most of the patients were able to taper their steroid dose and duration [47]. A second study mentioned in this systematic review examined four patients with the same profile. One patient’s negative result was deemed to be related to tumor progression rather than radiation necrosis, but the other three patients improved both clinically and radiologically and were able to discontinue steroids [37,50].

Thus, there is increasing evidence supporting the use of bevacizumab in the treatment of radiation necrosis.

## 7. Bevacizumab for Non-Tumor Indications and Miscellaneous

Wick and Kuker already highlighted the issue of vasogenic brain edema that can occur in patients suffering from brain tumors, further complicating the condition, including exacerbating the mass effect of the tumor, as well as neurological deterioration, as per Roth et al. [52]. Among the mechanisms involved in the pathophysiology that they highlight is the expression of VEGF by the tumor, specifically in those with significant peritumoral edema. Thus, bevacizumab, an anti-VEGF-A monoclonal antibody, would seem to be a promising solution [52,53]. Indeed, Vredenburgh et al. and Gerstner et al. demonstrated an important reduction in edema using bevacizumab [54,55].

In addition, several studies, as an aside, demonstrated that bevacizumab was effective in diminishing many tumor-associated symptoms. For instance, in a large review and meta-analysis on the use of bevacizumab in progressive pediatric low-grade glioma, Lu et al. attributed motor and endocrine improvements to be probably due to the easing of peritumoral edema thanks to bevacizumab [12]. Likewise, Liu et al. found that post-irradiation decline in DIPG patients was responsive to bevacizumab post-dexamethasone, and this was likely related to the decrease in edema or necrosis [50]. Alsahlawi et al. reported the case of a 14-year-old boy with an HGG in whom bevacizumab allowed for a decrease in vasogenic edema and weaning off steroids [56].

Bai et al. published a paper on the use of machine learning in predicting peritumoral edema response to bevacizumab based on imaging of the edema, in cases of metastatic brain tumors. The most influential factors in order were found to be edema volume, edema index, patient age, and tumor volume. They corroborated these findings both with multivariate analysis and logically, considering that larger edema reflects greater VEGF activity. This could have the potential to differentiate patients who are likely to respond from the ones who are not [57].

## 8. Safety and Toxicity of Bevacizumab

A common phrase among the many articles consulted was that the treatment was well tolerated [12,13,18,34,36,37,40,47,49,58]. Due to this high tolerability, some children were able to receive bevacizumab over a long period of time, even up to 24 cycles [58]. Likewise, the discontinuation rate due to toxicity was often reported as minimal [13].

Before further considering the safety and toxicity of bevacizumab, it is important to note that several of the studies consulted on the topic involved bevacizumab in combination with other medications. Therefore, attributing adverse events to a single agent can be difficult. For instance, Lu et al. described that chemotherapy-induced proteinuria is greater when bevacizumab is combined with another chemotherapy agent [12]. Also noteworthy is that the toxicities of bevacizumab can present differently in children than in adults. Nevertheless, the overall patterns remain similar [59].

There were, however, some toxicities that were identified as being specifically due to bevacizumab. Metts et al. observed grades 1-4 hypertension (maybe provide % of grade 3 and 4) due to bevacizumab in their phase I study [58]. In studying the use of bevacizumab in combination therapy for medulloblastoma, Levy et al. found hypertension due to bevacizumab in 38% of their patients [44].

Another important adverse event observed with the use of bevacizumab is proteinuria, which was the most common serious complication requiring medical intervention in a systematic review by Lu et al. [12]. In Levy et al.’s study, for example, proteinuria was observed in 22% of patients [44].

These two adverse events seem to be the consistently appearing toxicity profile associated with bevacizumab, as described by Calo et al., and as they found in previous literature [18]. Of note, while they are more frequent, they are also most often manageable [13], but have been equally responsible for the discontinuation of the medication, for example, in the study on NF2 schwannomas [40].

In adults, poor wound healing has been found to be associated with the use of bevacizumab [59]. Indeed, there have been some reports of delayed wound healing in children. However, in a PBTC study on recurrent CNS tumors treated with bevacizumab and irinotecan, Fangusaro et al. did not observe any cases of poor wound healing among the 92 children assessed [59]. Nevertheless, despite the sparse evidence in children, bevacizumab is still withheld perioperatively following adult guidelines [60,61].

Other adverse events include thromboembolic events, secondary amenorrhea, bone lesions and pain, fatigue, hyperglycemia, hyperkalemia, gastrointestinal toxicity, synovitis, and epistaxis, but to a lesser degree [12].

There are also some factors whose risk remains unknown. Imai et al. raised concerns regarding the effects of bevacizumab on fertility, given the important role of VEGF in follicular growth and ovarian function [62]. Zhukova et al. report two cases of adolescent, post-pubertal girls treated with bevacizumab who experienced amenorrhea with features of premature ovarian failure. Further research into these effects is necessary, as well as exploring the effect when administered to pre-pubertal patients. Without minimizing the uncomfortable and sometimes dangerous side effects, bevacizumab remains an overall tolerable and safe option for many cancers that would otherwise be disastrous, and a safer alternative to other known therapies [12,40].

## 9. Discussion and Future Directions

Given that many of the studies mentioned examined relatively small cohorts of patients, there is a common desire for further, larger, controlled, and more robust studies [12,13,37]. Also, many of the studies have been retrospective in nature. The experience of HERBY, ACNS0821, ACNS0822, and OZM-63 shows the importance of assessing new agents properly in large, collaborative trials.

Many of the studies also examined bevacizumab in conjunction with other medications, some of which, like irinotecan, have significant side effects and toxicities. It would therefore be interesting to evaluate the efficacy of bevacizumab as a monotherapy, given its tolerability and lack of major toxicities [18]. Nevertheless, further studies on combination therapies are still indicated and beneficial, such as in DIPG [36] and other CNS tumors [58]. These studies may also include novel combination therapies [13]. The combination of bevacizumab and targeted treatments like MEK and BRAF inhibitors has some preliminary evidence of being safe [63]. However, there has been no large study on the combination of these agents or the combination of Pan-RAF inhibitors with bevacizumab. Altogether, these future studies would help bridge gaps in the evidence and help standardize outcome measures, something that is lacking in much of the current literature [12,13,40].

Another common area that requires attention is the assessment and definition of response in patients treated with bevacizumab. In the adult setting, the RANO working group is tasked with developing objective response criteria [64]. They highlight the issue of bevacizumab, whereby when reducing vascular permeability, it becomes difficult to appreciate disease progression due to the emergence of non-enhancing tumor progression. Nearly 40% of cases demonstrate a stable-appearing contrast-enhancing disease, but non-enhancing disease increases on T2/FLAIR sequences [65].

Alternative imaging techniques, such as advanced MR and PET, can be of use in this case. Additionally, brain metrics may prove helpful for managing these patients. Thus, Ramakrishnan et al. conclude that a multimodal approach addressing the tumor, the patient, and treatments would be most important for assessing response [64].

In children, however, specific challenges exist, such as increased tumor heterogeneity and diversity. For this reason, the Response Assessment in Pediatric Neuro-Oncology (RAPNO) criteria were developed. These criteria do emphasize non-enhancing infiltrative disease, which, as Chukwueke et al. mentioned [65], is very relevant when using bevacizumab. RAPNO likewise modified other response criteria for the pediatric setting, and they provide specific criteria for different tumor classes (LGG, HGG, etc.) [64].

This is especially true when considering radiological responses compared to clinical responses. For example, Green et al. discussed the significant discordance between radiological and clinical changes in children with OPG [13]. Likewise, regarding NF2 schwannomas, both adults and children were found to have similar improvements in hearing, but tumor regression was less evident in children [40].

It may thus be an important future endeavor to examine clinical and quality of life changes to measure the global effect of bevacizumab and better establish its use.

Specifically, Barone and Rubin discuss defining response through laboratory biomarkers and imaging techniques [49]. This would be especially useful when biopsies, for example, are unavailable. Some of the lab techniques they mention include serum evaluations for pro-angiogenic factors, as well as angiogenesis-related biomarkers (VEGF, PDGF, PIGF). Continued production of markers of angiogenesis would indicate a poor response to the inhibition of neovascularization. Improved response definition through imaging includes more advanced MRI techniques and PET imaging. They postulate that the integration of a more enhanced monitoring of response would help time adjunctive interventions, avoid resistance, and increase efficacy.

Finally, optimization of the therapy will be an interesting area of future research. The optimal dosage and schedule for bevacizumab has not been clearly outlined for children, and doing so could render the treatment more effective and perhaps reduce cumulative toxicity [13,40], especially in those with prolonged treatment [40]. Furthermore, it will be very interesting to see the progression of machine learning and its utilization in guiding effective bevacizumab use, as described by Bai et al., in optimizing the therapy in those who will benefit and avoiding it in those who are unlikely to respond [57].

## 10. Conclusions

There is increasing evidence that bevacizumab has a role in pediatric neuro-oncology. Unfortunately, the paucity of properly designed clinical trials has been a major limiting factor for the recognition of the role of this agent in pediatric brain tumors. It is our hope that this review will clarify how far we have come with bevacizumab and inspire future research to further expand our knowledge of antiangiogenic agents, with the ultimate goal of improving patient care.
